# Effect of allogeneic pure platelet-rich plasma, sodium cloxacillin, and their combination for the treatment of subclinical mastitis in crossbred cows

**DOI:** 10.3389/fvets.2024.1432354

**Published:** 2024-08-22

**Authors:** Catalina López, Paulo Cesar Duque-Madrid, Alejandro Ceballos-Márquez, Jorge U. Carmona

**Affiliations:** ^1^Grupo de Investigación Patología Clínica Veterinaria, Departamento de Salud Animal, Universidad de Caldas, Manizales, Colombia; ^2^Grupo de Investigación Calidad de Leche y Epidemiología Veterinaria, Departamento de Producción Agropecuaria, Universidad de Caldas, Manizales, Colombia; ^3^Grupo de Investigación Terapia Regenerativa, Departamento de Salud Animal, Universidad de Caldas, Manizales, Colombia

**Keywords:** platelet-rich plasma, bovine, subclinical mastitis, gram-positive bacteria, growth factors, cytokines

## Abstract

**Introduction:**

Bovine subclinical mastitis (SCM) caused by Gram-positive bacteria is a major cause of economic loss in the dairy industry, exacerbated in situations where antimicrobial resistance is present. Pure platelet-rich plasma (P-PRP) may be a therapeutic alternative for SCM, when used alone or with antibiotics, such as sodium cloxacillin (SC). This study aimed 1) to evaluate the therapeutic efficacy of allogeneic P-PRP, SC, and their combination (P-PRP+SC) in cows with SCM caused by *Staphylococcus aureus* and by streptococci (*Staphylococcus aureus* and *S. dysgalactiae*); 2) to determine the concentrations of somatic cells (SCC), interleukin 1 beta (IL-1β), tumor necrosis factor-alpha (TNF-α) and TGF-β_1_ in milk samples of the cows.

**Methods:**

130 cows from 4 dairy herds completed the study, of which 40 cows were treated with P-PRP (10 mL), 28 cows with SC (5g), 36 with P-PRP+SC (10mL/5g), and 26 did not receive no treatment (negative control group, NCG).

**Results:**

The overall bacteriological cure was observed in 10/40 (25%) cows in the P-PRP group, 9/28 (32.14%) animals in the SC group, 26/36 (72.22%) cows in the P-PRP+SC group, and 10/26 (38.46%) animals in the NCG. SCM caused by *S. aureus* (82/130, 63.08%), was cured in 6/24 (25%) cows treated with P-PRP, 7/24 (29.2%) cows treated with SC, 8/16 (50%) animals treated with P-PRP+SC, and in 8/18 (44.4%) cows in NCG. For SCM caused by the streptococci (48/130, 36.91%), the cure was achieved in 4/12 (33.3%) cows treated with P-PRP, 2/4 (50%) cows treated with SC, 18/20 (90%) cows treated with P-PRP+SC, and in 2/8 (25%) cows of the NCG. SCC was significantly (*p* < 0.001) affected by the treatment, herd, cure, bacteria group, and number of calvings factors. IL-1β milk concentrations were significantly (*p* < 0.001) influenced by treatment and farm factors, and the interaction between these factors. TNF-α milk concentrations were significantly (*p* < 0.001) influenced by time factor. TGF-β_1_ milk concentrations were significantly affected by the time and cure factors.

**Conclusion:**

The combination of P-PRP and SC showed the best therapeutic response (90%) against bovine SCM caused by streptococci. However, none of the treatments showed an effective therapeutic response against *S. aureus*.

## Introduction

Subclinical mastitis (SCM) is one of the leading causes of economic losses for dairy producers worldwide. Each year, producers lose income due to reduced milk production from affected cows and changes in milk composition, particularly due to a significant increase in somatic cell count (SCC) (1, 2). Furthermore, when this type of problem is detected, usually through a milk quality program, the owners are forced to treat the affected cows with antibiotics, resulting in further costs for the treatment and the withdrawal time, and additional losses due to discarded milk (3). On the other hand, in many cases, antibiotic treatment does not work, so the producer’s effort to treat the cows results in additional economic losses (4).

Currently, despite measures aimed at preventing SCM, this disease continues to occur constantly in dairy herds, raising the question of whether conventional antibiotics should continue to be used to treat this disease (5–8). The answer is not simple, as there are policies, particularly in the United States of America and Europe, that are attempting to phase out the use of antibiotics for treating SCM in dairy cows, but this is not always easy (8, 9). It has not yet been possible to develop therapeutic alternatives that are as effective as antibiotics and at the same time free of residues and the development of microbial resistance to this type of drug (10). These last two factors are of great concern to public health authorities around the world (5–8). Therefore, greater efforts should be made to develop new products to replace antibiotics in the dairy market (8) or to enhance the therapeutic efficacy of approved antibiotics used for bovine mastitis.

Sodium cloxacillin (SC) is a β-lactam antibiotic commonly used to treat clinical mastitis (CM) and SCM in cows during lactation or for dry-off therapy at the end of lactation. This antibiotic is an isoxazoyl penicillin that is beta-lactamase stable and therefore may be effective against *S. aureus* strains that produce this enzyme (11). Although, β-lactamase-resistant penicillins, such as SC, are considered the first-line antimicrobials for the treatment of bovine mastitis caused by *S. aureus* due to the susceptibility of this bacterium to this group of antibiotics (12), a similar situation does not exist for streptococci causing mastitis where antimicrobial resistance to SC has been reported in 53.8% of bacterial isolates causing SCM (13). Therefore, new therapeutic alternatives should be proposed to either replace the antibiotics used in the treatment of bovine mastitis or enhance the therapeutic effect of these substances.

Platelet-rich plasma (PRP) is a hemocomponent rich in growth factors, such as transforming growth factor-beta 1 (TGF-β_1_) and platelet-derived growth factor BB (PDGF-BB), cytokines, and chemokines, such as platelet factor-4 (PF-4), which have various anti-inflammatory, regenerative, and bacteriostatic effects. According to the literature reviewed, two types of PRP have been used for the treatment of bovine mastitis. One of them was a pure PRP (P-PRP), which was characterized by negligible concentrations of white blood cells (WBCs) and moderate concentrations of platelets (14), which was evaluated in cows with SCM caused by Gram-positive bacteria (15). On the other hand, a PRP rich in leukocytes (L-PRP) and with higher platelet concentrations was evaluated in cows with clinical mastitis (CM) produced by Gram-positive and Gram-negative bacteria (16). The results of both studies were encouraging (15, 16); however, further research is needed to compare the effect of PRP, alone or in combination, with other antibiotics more commonly used to treat bovine mastitis, such as sodium cloxacillin (SC).

We present the results of a randomized controlled clinical trial that was conducted in crossbred cows with SCM caused by Gram-positive bacteria, evaluating the effect of an intramammary (IMM) preparation of P-PRP versus a suspension of SC and a combination of both products (P-PRP + SC). Additionally, a group of cows with the same disease that was not treated was included to evaluate the spontaneous cure of the IM infection (IMMI). The objectives of the study were (1) to determine the degree of bacteriological cure in the experimental groups and (2) to evaluate the effect of these substances on the milk concentration of somatic cells, proinflammatory cytokines [interleukin 1beta (IL-1b) and tumor necrosis factor-alpha (TNF-α)] and growth factors (TGF-β_1_ and PDGF-BB).

## Materials and methods

This study was approved by the Institutional Committee on Animal Experimentation. The experiments were conducted in accordance with the National Research Council’s Guide for the Care and Use of Laboratory Animals, the US Institute for Laboratory Animal Research, and the Colombian Animal Welfare Guidelines. In addition, this study was conducted in accordance with the ARRIVE guidelines. It is important to clarify that the animals included in this study were from conventional farms and were never euthanized for research purposes.

### Herds and animals

Initially, nine herds located in the low-tropical regions of the department of Caldas, Colombia, were included in the study. From these herds, bacteriological screening was performed on 650 milking animals to detect at least 160 cows with SCM according to the inclusion criteria established in the study. The inclusion criteria for cows were that they had no health problems other than SCM, had a clinically normal udder with no deformed or blind teats, were in the 1–5 calving range, had not been treated with antibiotics and anti-inflammatories in the previous 30 days, and had not had suffered an episode of CM in the current lactation.

### Blood collection and preparation of P-PRP

The P-PRP used in this study was obtained from six Blanco Orejinegro heifers aged 24–36 months with an average body weight of 400 ± 40 kg. Whole blood from each heifer was collected by aseptic venipuncture and placed in double transfusion bags (Terumo Double Bag CPDA-1, NJ, United States) according to the procedures described in a previous study (15). All blood donor animals were closely monitored for anemia (by weekly full-cell hemogram) and any other health problems. Heifers in the P-PRP and P-PRP + SC groups were bled every 2 weeks until treatment was completed.

The blood bags were immediately centrifuged in a stationary centrifuge (RotoSilenta 630 RS, Hettich, Tuttlingen, Germany) at 698 g/6 min. The plasma fraction (P-PRP), including the WBC layer, was then transferred from each blood bag to the satellite plasma bag. The P-PRP was dispensed into 10-mL syringes. Each dose of P-PRP was accompanied by a sterile reaction tube containing 1 mL of calcium gluconate, which was added to the P-PRP immediately before intramammary infusion (15).

### Assessment of cells and mediators in P-PRP

A total of 30 10-ml P-PRP syringes were randomly selected for automated total cell counting (Celltac α MEK-6450, Nihon Kohden, Tokyo, Japan). The samples were then activated with calcium gluconate in a 1:10 ratio and incubated at 37°C for 3 h to allow the release of platelet mediators and platelet gel formation. After this time, P-PRP supernatants were collected and growth factors (TGF-β_1_ and PDGF-BB) and the chemokine platelet factor-4 (PF-4) were measured.

### Design of the study and power sample calculation

This randomized clinical trial was designed to determine the effect of three treatments (P-PRP, SC, and P-PRP + SC) in cows with SCM caused by Gram-positive bacteria. An NCG composed of cows that were not treated was also included. The experimental treatment groups were as follows: (a) P-PRP (10 mL, IMM), (b) SC (5 g, equivalent to 200 mg of the antibiotic, IMM), and (c) P-PRP + SC (10 mL of P-PRP plus 5 g of CS, IMM). All treatments were administered in a blinded fashion. The first treatment was administered 4 days after the diagnosis of IMMI. Each quarter of each cow, for each of the experimental groups (P-PRP, Ab, and P-PRP + Ab) was treated after milking and teat disinfection with the administration of three doses of each product evaluated at 24 h intervals for 3 consecutive days.

The sample size for this clinical trial was calculated *a priori* taking into account a β value of 0.8 and a α value of 0.05, which were based on the previous data from another clinical trial (15). Accordingly, the n calculated for each group was a minimum of 28 cows per experimental group and a total of 112 cows for the study. It is important to clarify that 160 cows were selected at the beginning of the clinical trial to reduce the risk of withdrawal of animals (and even farms) from each of the experimental groups evaluated during the development of the study.

### Bacteriological workflow and declaration of the intramammary infection

Using an aseptic technique, two composite milk samples were collected from each cow before milking on the first visit to the farm (day 0) to select potential animals for inclusion in the study. The milk samples were refrigerated and sent to our laboratories for analysis. One sample was used for automated somatic cell count (SCC) (Fossomatic, Foss, Hillerød, Denmark). SCC results in milk were expressed as a linear scale (LSSCC) in thousands/ml to normalize the data distribution.

Based on the arithmetic SCC results (≥ 100,000 cells/mL in primiparous cows and ≥ 200.000 cells/mL in multiparous cows), the second milk sample was used for bacteriological cultures and for the determination of milk cytokines by ELISA [IL-1b (Bovine IL-1β ELISA Reagent Kit, Thermo Fisher Scientific Inc., Waltham, MA, United States) and TNF-a (Bovine TNF-alpha DuoSet ELISA, R&D Systems, Minneapolis, MN, United States)] and GFs [TGF-β_1_ (Human TGF-β1 DuoSet, DY240E, R&D Systems, Minneapolis, MN, United States)] and PDGF-BB (Human PDGF-BB DuoSet, DY220, R&D Systems, Minneapolis, MN, United States), as performed in a previous study (15).

The microbiological workflow for the detection and declaration of staphylococcal and streptococcal infections was performed according to the protocols established by the National Mastitis Council (17). Cultures with more than two bacterial species were considered contaminated and not indicative of IMMI.

Intramammary infection was declared when a composite milk sample had an SCC above the established cutoff points for primiparous and multiparous cows and the microbiological culture was positive for any major Gram-positive mammary pathogen, such as *S. aureus*, *S. agalactiae*, *S. uberis*, and *S. dysgalactiae* (15).

### Cure determination procedure

Following treatment of the animals, pooled milk samples were collected on days 21 and 22. Microbiological analysis, SCC, cytokine, and GF assays were performed on each milk sample as described above. Cure was defined in cows that were infected at baseline and where the organism present was not isolated in the two post-treatment samples.

The cure was defined at the cow level, and the risk of cure was statistically evaluated for the treatment group at 22 days. A reduction in LSSCC (cells/ml milk) on day 22 was defined as a possible cure for the two post-treatment samples (obtained on days 21 and 22) compared to the first value obtained on day 0, provided that bacteriological cultures were negative. Changes in mediator concentrations in milk were also used as criteria for cure and to evaluate mammary gland inflammation.

### Statistical analysis

Data were analyzed using the JASP statistical software (JASP 0.17.1 (Intel) University of Amsterdam, The Netherlands). Data for each variable were presented using descriptive statistics. Statistical analysis was performed using generalized linear mixed models (GLMMs) according to the type of the response variable (either dichotomous or continuous). The main classifying factors analyzed for the dichotomous GLMM evaluating the cure of the cows (0 = not cured; 1 = cured) were as follows: treatment (with four levels: P-PRP, SC, P-PRP + SC, and NCG), herd (with four levels), number of calvings (with four levels), and LSSCC/mL at day 0. A binomial family GLMM and a logit link function were used to determine differences between the overall (considering all pathogens) probability of cure (=1). In addition, individual GLMMs were performed to determine the probability of cure of SCN caused by *S. aureus* and the non-agalactiae streptococci (*S. uberis* and *S. dysgalactiae*).

For the GLMMs in which the response variables were of a continuous numerical nature Gaussian family distributions and identity link functions were used; in addition to the classification factors mentioned above, time (with two levels: day 0 and day 22), cure (with two levels: 0 = not cured, 1 = cured), and type of bacteria (with two levels: *S. aureus* and streptococci group) were taken into account to develop the statistical models.

The cow was declared as a random effect variable in all models. The best models were built using only statistically significant response variables (*p* < 0.05). Similarly, the best models were selected by the value of the Akaike information criterion (AIC) obtained for each model, assessing the individual effect of each response variable according to the Anderson and Burnham criteria (18). GLMMs were then performed including the study of interactions between the highly significant factors previously identified. When statistically significant differences were found for the factors or the interaction between them, Scheffé *post-hoc* tests were performed. A *p* < 0.05 was considered statistically significant for all tests performed.

## Results

### Herds and animals

Of the 160 cows enrolled at the beginning of the study, only 130 cows completed the clinical trial. 30 cows from five of the nine herds initially enrolled did not complete the clinical trial, leaving only four herds analyzed in the study. The groups evaluated were as follows: P-PRP, SC, PRP + SC, and NCG had 40, 28, 28, 36, and 26 cows, respectively. The cows included in the study had a mean age of 59.52 (±27.38) months (range 26.30–145.2 months) with a mean milk production of 15.55 (±5.46) L/day (range 5–27 L/day) and 2.77 (±1.20) calves on average.

### Cell and mediator concentrations in P-PRP

The P-PRP used in the clinical trial had a concentration of 260 (±40) × 10^3^ platelets/μL and 0.23 (±0.012) leukocytes/μL. After activation of the P-PRP with calcium salts, an average concentration of 56.03 (±13.13) pg/mL of PDGF-BB, 9345.23 (±800.91) pg/mL of TGF-β_1_, and 5.10 (±1.66) pg/mL of PF-4 was measured in the supernatants derived from this hemocomponent.

### Intramammary infections and treatment assignment

At baseline, 82 out of 130 (63.06%) cows had SCM caused by *Staphylococcus aureus*, 36 out of 130 (27.68%) cows were infected by *Streptococcus uberis*, and 12 out of 130 (9.22%) animals were infected by *Streptococcus dysgalactiae*. The baseline LSSCC in milk was 15.97 (±1.58) cells/mL. Table 1 shows the distribution of cows by etiological agent and herd, and Table 2 shows the distribution of cows by assigned experimental group and etiological agent of SCM.

**Table 1:** Causative agent of subclinical mastitis (SCM) in the cows and distribution of infected cows per herd.

Pathogen	Herd	Cows (*n*)
*Staphylococcus aureus*	1	36
	2	20
	3	18
	4	8
Subtotal		82
*Streptococcus uberis*	1	0
	2	10
	3	20
	4	6
Subtotal		36
*Streptococcus dysgalactiae*	1	0
	2	4
	3	2
	4	6
Subtotal		12
Total		130

**Table 2:** Causative agent of subclinical mastitis in the study cows and distribution of cows by assigned experimental group.

Pathogen	Group	Cows (*n*)
*Staphylococcus aureus*	P-PRP	24
	SC	24
	P-PRP + SC	16
	NCG	18
Subtotal		82
*Streptococcus uberis*	P-PRP	12
	SC	4
	P-PRP + SC	16
	NCG	4
Subtotal		36
*Streptococcus dysgalactiae*	P-PRP	4
	SC	0
	P-PRP + SC	4
	NCG	4
Subtotal		12
Total		130

P-PRP, Pure platelet-rich plasma; SC, Sodium cloxacillin; NCG, Negative control group.

### Overall bacteriological cure

**Figure 1:**
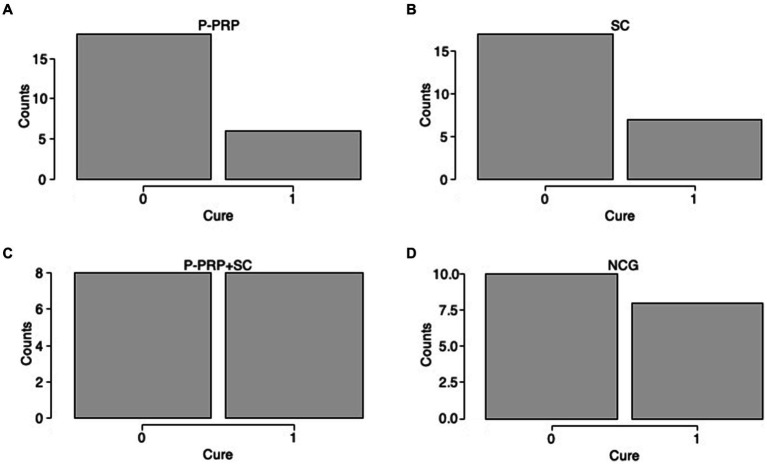
Distribution plots of the percentage of cows cured in the study by treatment factor. Acronyms as in Tables 2 and 4. 0= infected; 1= cured.

The overall bacteriological cure was observed in 10 out of 40 (25%) cows in the P-PRP group, nine out of 28 (32.14%) animals in the SC group, 26 out of 36 (72.22%) cows in the P-PRP + SC group, and 10 out of 26 (38.46%) animals in the negative control group (Figures 1A–D). The exploratory GLMM was significantly influenced by the fixed-factors treatment, herd, and LSSCC/mL at day 0 (Table 3). Subsequently, the interaction model was significantly affected by the fixed-factors treatment, herd, and LSSCC/mL count at day 0 and for the interactions between treatment × herd and treatment × LSSCC/mL at day 0 (Table 3).

**Table 3:** GLMMs evaluating the effect of fixed factors and their interaction on the probability of overall cure in the cows of the study.

GLMM type	Fixed factor	df	ChiSq	*p*
Exploratory	Intercept	1	10.390	< 0.001
	Treatment	4	18.778	< 0.001
	Herd	3	7.985	0.046
	Parity (*n*)	4	0.586	0.387
	LSSCC/mL (day 0)	1	9.032	< 0.001
Interaction	Intercept	1	13.539	< 0.001
	Treatment	3	8.355	0.039
	Herd	3	0.000	1.000
	LSSCC/mL (day 0)	1	12.235	< 0.001
	Treatment × herd	9	36.293	< 0.001
	Treatment × LSSCC/mL (day 0)	3	9.453	0.024

LSSCC, Linear scale of somatic cell count. Other acronyms as in Table 2.

**Figure 2:**
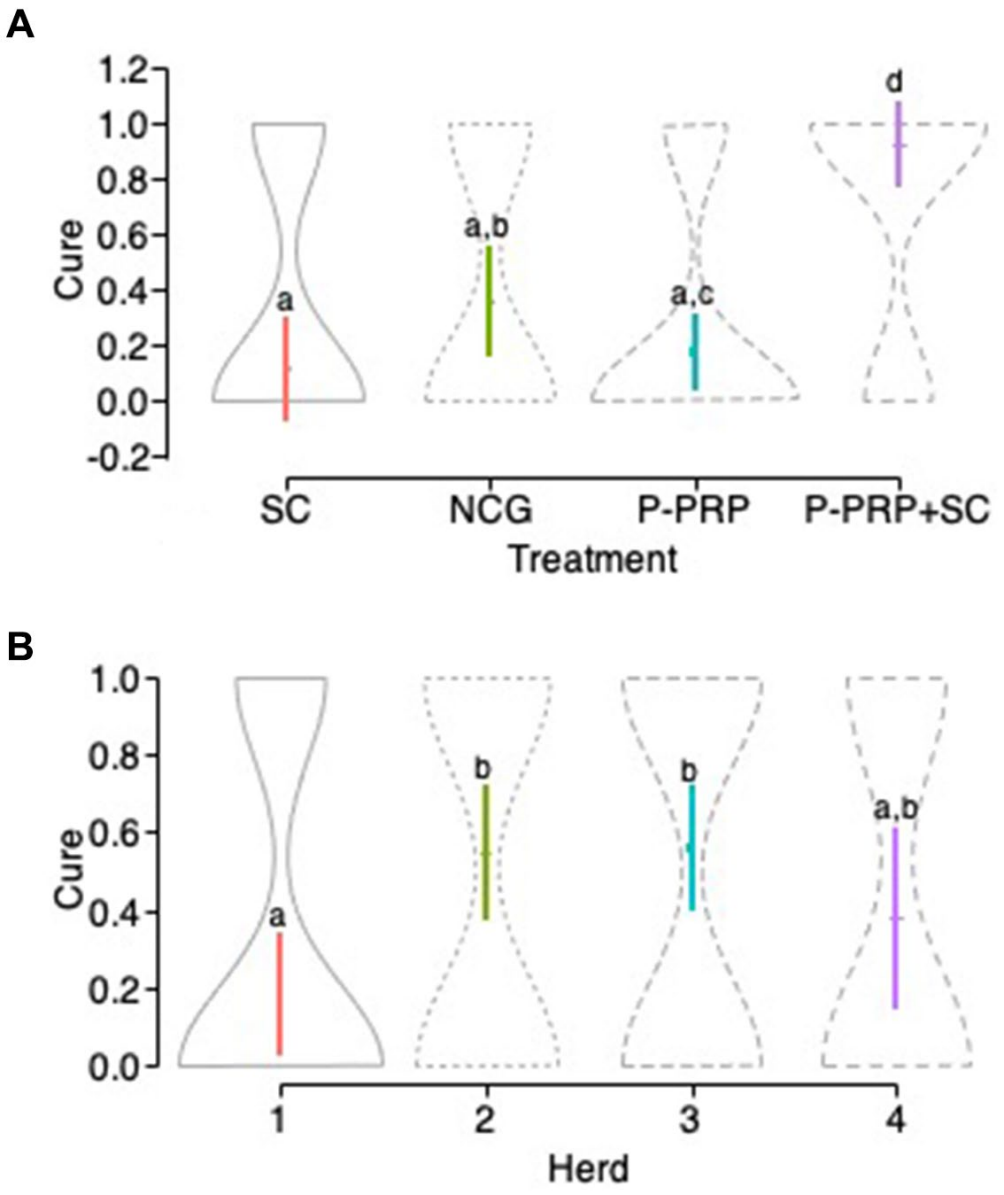
Violin plots showing the confidence intervals (95%) for the mean of the treatment factor **(A)** and the herd factor **(B)** in cows with SCM. ^a–d^Different lower-case letters represent statistically significant differences between groups for the Scheffé test. Acronyms as in Tables 2, 4.

Regarding the treatment response to infection, a significant (*p* = 0.001) therapeutic response was observed in the group of cows treated with the P-PRP + SC combination compared to the other two treatments evaluated and to the NCG. On the other hand, the cure results of the group of cows treated with SC were similar to those of the groups of cows treated with P-PRP and the NCG. In addition, a significant difference (*p* = 0.021) was observed between the negative control group of cows treated with P-PRP (Figure 2A).

Regarding the effect of the herd in the general infection model, it was observed that herd 1 had a significantly lower probability of cure than herds 2 (*p* = 0.001) and 3 (*p* = 0.001) and did not differ from herd 4. When analyzing the significant interaction between treatment and herd factors (Table 3), it was generally observed that the groups of animals treated with P-PRP in herds 2, 3, and 4 responded better than the group treated with the same product in herd 1. Similarly, the cows treated with SC showed significant therapeutic responses in herds 1, 2, and 3 and low cure rates in herd 4 (Figure 2B).

Herds 2 and 3 showed high cure rates in the P-PRP + SC groups, while herds 1 and 4 showed variable responses to the same treatment. Concerning the NCG, cows from herd 2 had the least healing compared to cows from the other herds, where many of which healed spontaneously (see Table 4 for specific statistically significant differences).

**Table 4:** Estimated marginal means for overall cure about the interaction between treatment and herd factors.

				95% CI			
Herd	Treatment	Estimate	SE	Inferior	Superior	*z*	*p*
1	SC	0.041	0.047	0.004	0.308	−2.636	0.008
2	SC	0.189	0.128	0.043	0.546	−1.740	0.082
3	SC	0.197	0.146	0.038	0.601	−1.519	0.129
4	SC	0.105	0.104	0.013	0.506	−1.939	0.053
1	NCG	0.159	0.102	0.040	0.458	−2.179	0.029
2	NCG	0.507	0.162	0.224	0.785	0.041	0.967
3	NCG	0.519	0.151	0.247	0.780	0.125	0.901
4	NCG	0.342	0.172	0.104	0.699	−0.857	0.391
1	P-PRP	0.066	0.061	0.010	0.330	−2.673	0.008
2	P-PRP	0.276	0.139	0.089	0.598	−1.388	0.165
3	P-PRP	0.286	0.124	0.109	0.568	−1.510	0.131
4	P-PRP	0.162	0.116	0.035	0.509	−1.916	0.055
1	P-PRP + SC	0.809	0.180	0.301	0.977	1.238	0.216
2	P-PRP + SC	0.958	0.056	0.591	0.997	2.219	0.026
3	P-PRP + SC	0.960	0.052	0.628	0.997	2.345	0.019
4	P-PRP + SC	0.921	0.097	0.460	0.994	1.840	0.066

SE, Standard error; CI, Confidence interval. Other acronyms as in Table 2.

Regarding the interaction of the fixed factors treatment × LSSCC/mL at day 0, it was observed that the cows that responded significantly to the treatment with P-PRP and P-PRP + SC had LSSCC/mL at day 0 equal to or lower than 15.97 (cells/mL), while for the SC treatment group, significant responses were observed for LSSCC/mL at day 0 between 14.36 and 15.97 cells/mL. On the other hand, cows in the NCG were not affected by LSSCC/mL at day 0 (see Table 5 for exact statistical conclusions).

**Table 5:** Estimated marginal means for overall cure about the interaction between treatment factor × LSSCC/mL at day 0.

				95% IC			
LSSCC/mL	Treatment	Estimate	SE	Inferior	Superior	*z*	*p*
14.372	SC	0.021	0.033	9.053 × 10^−4^	0.338	−2.375	0.018
15.965	SC	0.115	0.092	0.022	0.434	−2.255	0.024
17.558	SC	0.441	0.150	0.192	0.723	−0.392	0.695
14.372	NCG	0.193	0.124	0.048	0.532	−1.798	0.072
15.965	NCG	0.365	0.117	0.176	0.607	−1.099	0.272
17.558	NCG	0.579	0.188	0.232	0.862	0.412	0.680
14.372	P-PRP	0.216	0.124	0.061	0.537	−1.756	0.079
15.965	P-PRP	0.176	0.093	0.058	0.428	−2.417	0.016
17.558	P-PRP	0.142	0.121	0.023	0.538	−1.806	0.071
14.372	P-PRP + SC	0.552	0.150	0.273	0.802	0.345	0.730
15.965	P-PRP + SC	0.928	0.082	0.536	0.993	2.078	0.038
17.558	P-PRP + SC	0.993	0.019	0.471	1.000	1.914	0.056

Acronyms as in Tables 2, 4.

### Specific bacteriological cure

Regarding the response to the different treatment groups for each of the isolated bacteria, it was decided to analyze the effect of these against *S. aureus* and the streptococci group consisting of *S. uberis* and *S. dysgalactiae*, as the latter bacterium had a very low representation as the etiological agent of the SCM cases (*n* = 12 out of 130) found in the study, and even in herd 1, no cases were reported.

**Figure 3:**
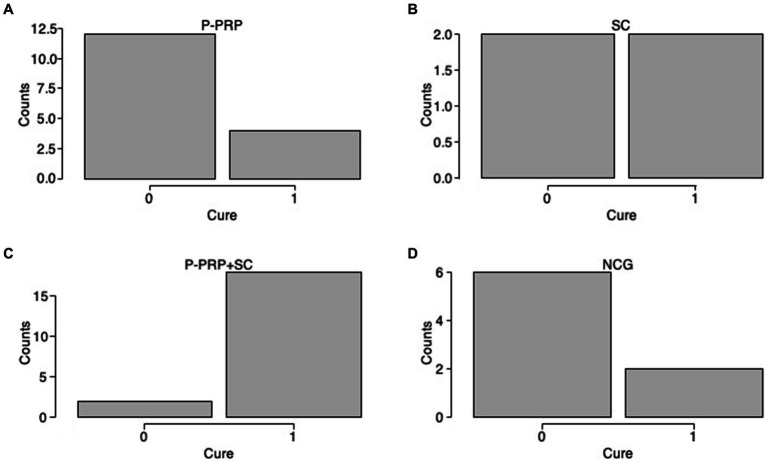
Distribution plots of the *Staphylococcus aureus* cure rate of cows with SCM by treatment factor. Acronyms as in Tables 2 and 4. 0= infected; 1= cured.

**Figure 4:**
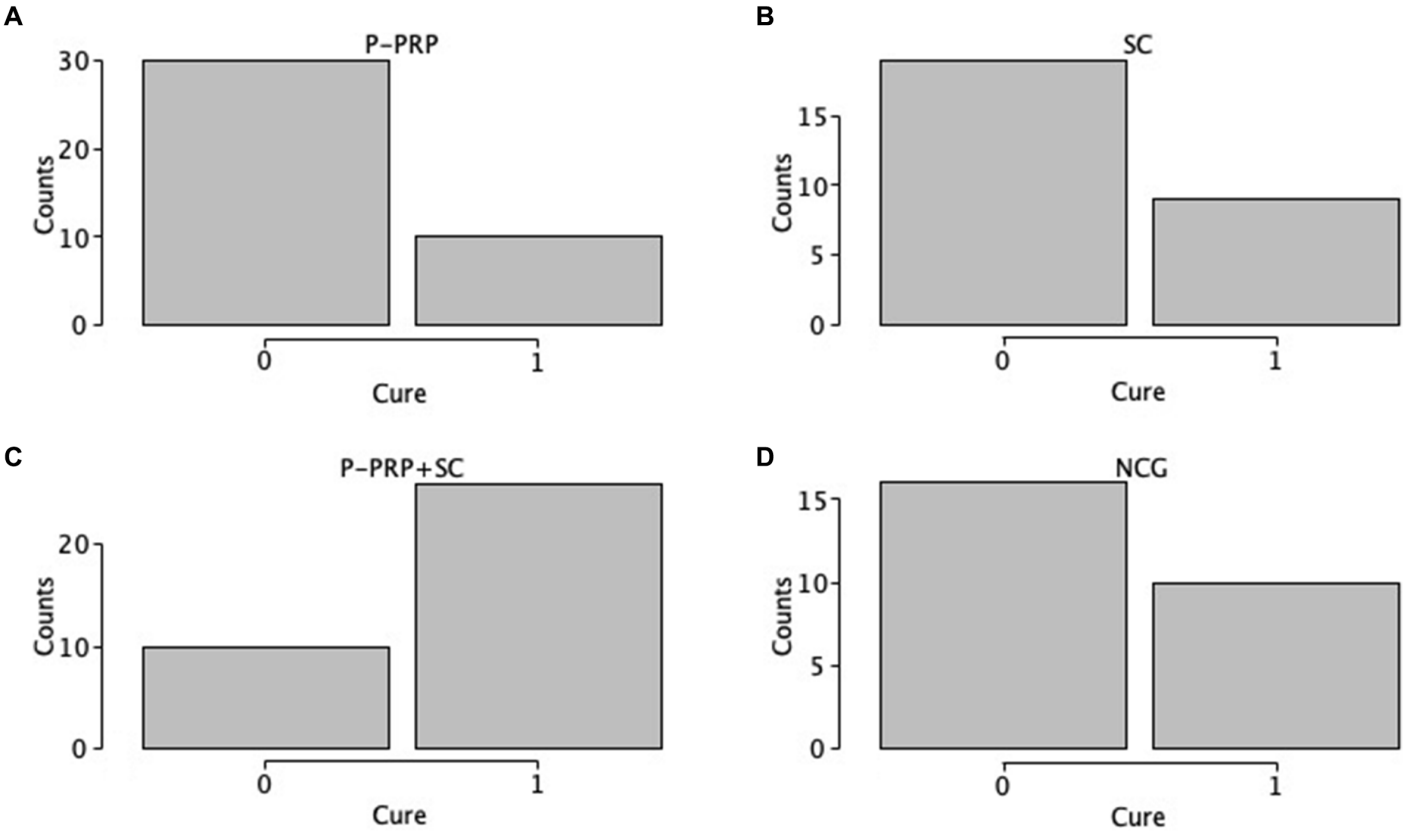
Distribution plots of the cure rate for the streptococci group (*S. uberis* and *S. dysgalactiae*) in the cows with SCM according to the treatment factor. Acronyms as in Tables 2 and 4. 0= infected; 1= cured.

For *S. aureus*-induced SCM (82 out of 130, 63.08%), the cure was observed in six out of 24 (25%) cows treated with P-PRP, seven out of 24 (29.2%) cows treated with SC, eight out of 16 (50%) animals treated with P-PRP + SC, and eight out of 18 (44.4%) cows of the NCG (Figures 3A–D). On the other hand, SCM produced by the streptococcus group (48 out of 130, 36.91%) was cured in four out of 12 (33.3%) P-PRP-treated cows, two out of 4 (50%) animals treated with SC, 18 out of 20 (90%) cows treated with P-PRP + SC, and two out of 8 (25%) cows of the NCG (Figures 4A–D).

Regarding the response to treatment, evaluating only the population of cows infected with *S. aureus*, the exploratory GLMM revealed only a significant effect of LSSCC/mL at day 0 as a factor associated with the cure of cows infected with this bacterium (Table 6). In general, it was observed that cows with LSSCC/mL at day 0 equal to or less than 14.25 cells/mL had a significantly higher cure rate than cows with LSSCC/mL above this concentration of somatic cells in milk (Table 7).

**Table 6:** GLMMs evaluating the effect of fixed factors and their interaction on the probability of cure of the SCM caused by *S. aureus* in the cows of the study.

GLMM type	Fixed factor	df	ChiSq	*p*
Exploratory	Intercept	1	10.939	< 0.001
	Treatment	3	4.272	0.234
	Herd	3	1.975	0.578
	Parity (*n*)	4	1.636	0.802
	LSSCC/mL (day 0)	1	9.372	0.002

Acronyms as in Tables 2, 4.

**Table 7:** Estimated marginal means for cure response against SCM caused by *S. aureus* about LSSCC/mL at day 0.

			95% IC			
LSSCC/mL	Estimate	SE	Inferior	Superior	*z*	*p*
14.245	4.506 × 10−4	0.001	1.890 × 10−6	0.097	−2.758	0.006
16.021	0.217	0.321	0.007	0.919	−0.678	0.498
17.798	0.994	0.022	0.093	1.000	1.358	0.174

Acronyms as in Tables 2–4.

**Figure 5:**
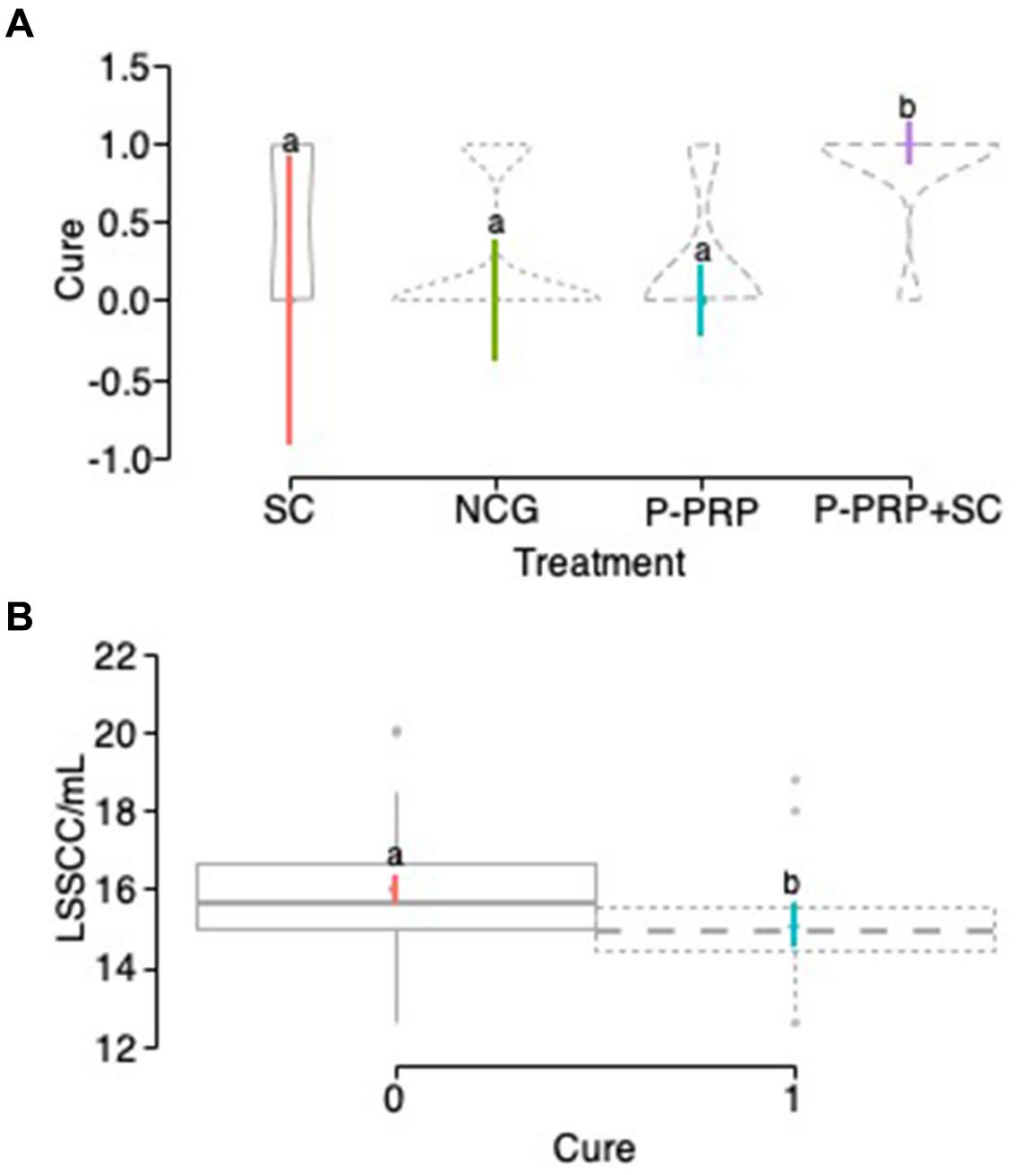
**(A)** Violin plots showing the confidence intervals (95%) for the mean of the treatment factor in cows with SCM caused by the group of streptococci (*Streptococcus uberis* and *Streptococcus dysgalactiae*). **(B)** Box plots showing the confidence intervals (95%) for the mean milk LSSSC/mL in the cows with SCM according to the cure factor (0 = infected; 1 = cured). ^a,b^Different lower-case letters represent statistically significant differences between groups for the Scheffé test. Acronyms as in Tables 2, 4.

The exploratory GLMM of individual fixed effects in the group of streptococci-infected cows showed that only the treatment factor had a significant effect on the model (Table 8). Regarding the treatment effect, it was observed that the combination of P-PRP + SC significantly increased (*p* = 0.005) the probability of cure of SCM produced by the streptococcal group compared to the other treatments. Similarly, no significant difference was found between the cows treated with SC and P-PRP and those animals from the NCG in terms of the probability of cure of this type of SCM (Figure 5A).

**Table 8:** GLMMs evaluating the effect of fixed factors and their interaction on the probability of cure of the SCM caused by *S. aureus* in the cows of the study.

GLMM type	Fixed factor	df	ChiSq	*p*
Exploratory	Intercept	1	0.022	0.882
	Treatment	3	24.187	< 0.001
	Herd	3	0.081	0.960
	Parity (*n*)	4	0.602	0.963
	LSSCC/mL (day 0)	1	0.041	0.839

Acronyms as in Tables 2, 4.

### Cellular and biochemical changes in milk

#### Somatic cell counts

In the exploratory model, LSSCC/mL was significantly affected by the fixed effects of treatment, herd, cure, bacteria group, and number of calvings, while the time factor did not affect this response variable (Table 9). However, in the interaction model, there was no significant effect of treatment, but there was a significant effect of the cure factor (Table 9; Figure 5B).

**Table 9:** GLMMs evaluating the effect of fixed factors and their interaction on the LSSCC/mL in the milk of the cows of the study.

GLMM type	Fixed factor	df	ChiSq	*p*
Exploratory	Intercept	1	584.100	< 0.001
	Treatment	3	209.637	< 0.001
	Time	1	0.044	0.834
	Herd	3	82.261	< 0.001
	Parity (*n*)	4	57.378	< 0.001
	Bacteria group	1	106.852	< 0.001
	Cure	1	393.181	< 0.001
Interaction	Intercept	1	146.859	< 0.001
	Treatment	3	2.716	0.438
	Cure	1	4.647	0.031
	Herd	3	2.833	0.418
	Bacteria group (BG)	1	0.279	0.598
	Treatment × cure	3	1.284	0.733
	Treatment × herd	9	5.355	0.802
	Treatment × BG	3	1.962	0.580

Acronyms as in Tables 2–4.

#### Mediator concentration in milk

IL-1β milk concentrations were significantly influenced in the exploratory model by the fixed-factors treatment, herd, and LSSCC/mL, while factors such as time, number of calving, cure, and type of bacteria did not influence the model (Table 10). Subsequently, in the interaction model, a significant effect was observed for the treatment, farm factors, and the interaction between treatment and herd factors, while the LSSCC/mL factor did not affect the model (Table 10).

**Table 10:** GLMMs evaluating the effect of fixed factors and their interaction on the interleukin 1 beta (IL-1b pg./mL) concentrations in the milk of the cows of the study.

GLMM type	Fixed factor	df	ChiSq	*p*
Exploratory	Intercept	1	6.052	0.014
	Treatment	3	33.034	< 0.001
	Time	1	0.513	0.474
	Herd	3	61.863	< 0.001
	Parity (*n*)	4	6.431	0.169
	LSSCC/mL	1	12.674	<0.001
	Cure	1	10.715	0.100
	Bacteria group (BG)	1	0.108	0.743
Interaction	Intercept	1	0.628	0.428
	Treatment	3	71.893	< 0.001
	Herd	3	49.701	< 0.001
	LSSCC/mL	1	0.456	0.500
	Treatment × herd	9	90.118	< 0.001
	Treatment × LSSCC/mL	3	5.291	0.152

Acronyms as in Tables 2–4.

**Figure 6:**
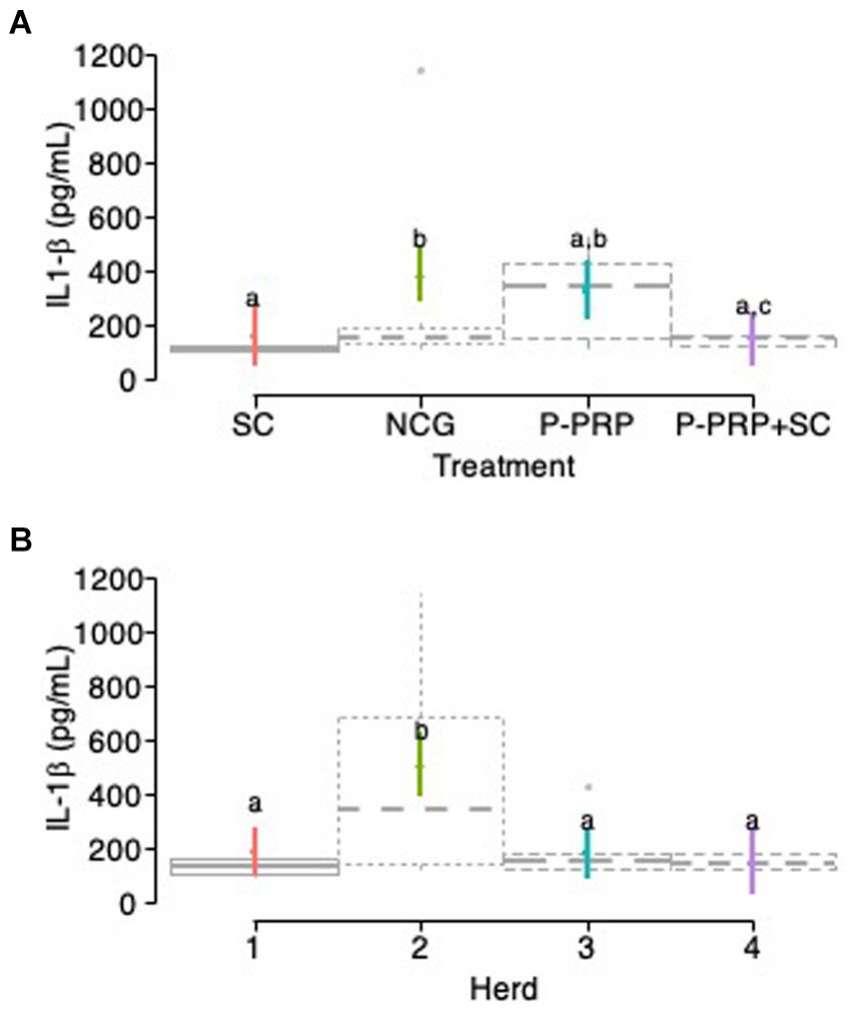
Box plots showing the confidence intervals (95%) for the mean concentrations (pg/mL) of interleukin 1 beta (IL-1β) in the milk of the study cows according to the treatment factor **(A)** and the herd factor **(B)**. ^a,b^Different lower-case letters represent statistically significant differences (<0.001) between the groups for the Scheffé test. Acronyms as in Tables 2, 4.

When evaluating the effect of treatment on total IL-1β concentrations (pg/mL) in milk, it was observed that cows treated with SC and P-PRP + SC had the lowest concentrations of this inflammatory cytokine than the other groups evaluated. However, IL-1β concentrations were similar between CS- and P-PRP-treated groups, while concentrations of this mediator were significantly higher in the NCG than SC (*p* = 0.004) and P-PRP + SC (*p* = 0.001) groups. Mediator concentrations were significantly (*p* = 0.029) higher in the milk of the P-PRP-treated group of cows than the P-PRP + SC-treated cows (Figure 6A). On the other hand, when the herd effect was evaluated, it was observed that milk concentrations of IL-1β were significantly higher (*p* = 0.001) in herds 2 than in herds 1, 3, and 4 (Figure 6B).

**Figure 7:**
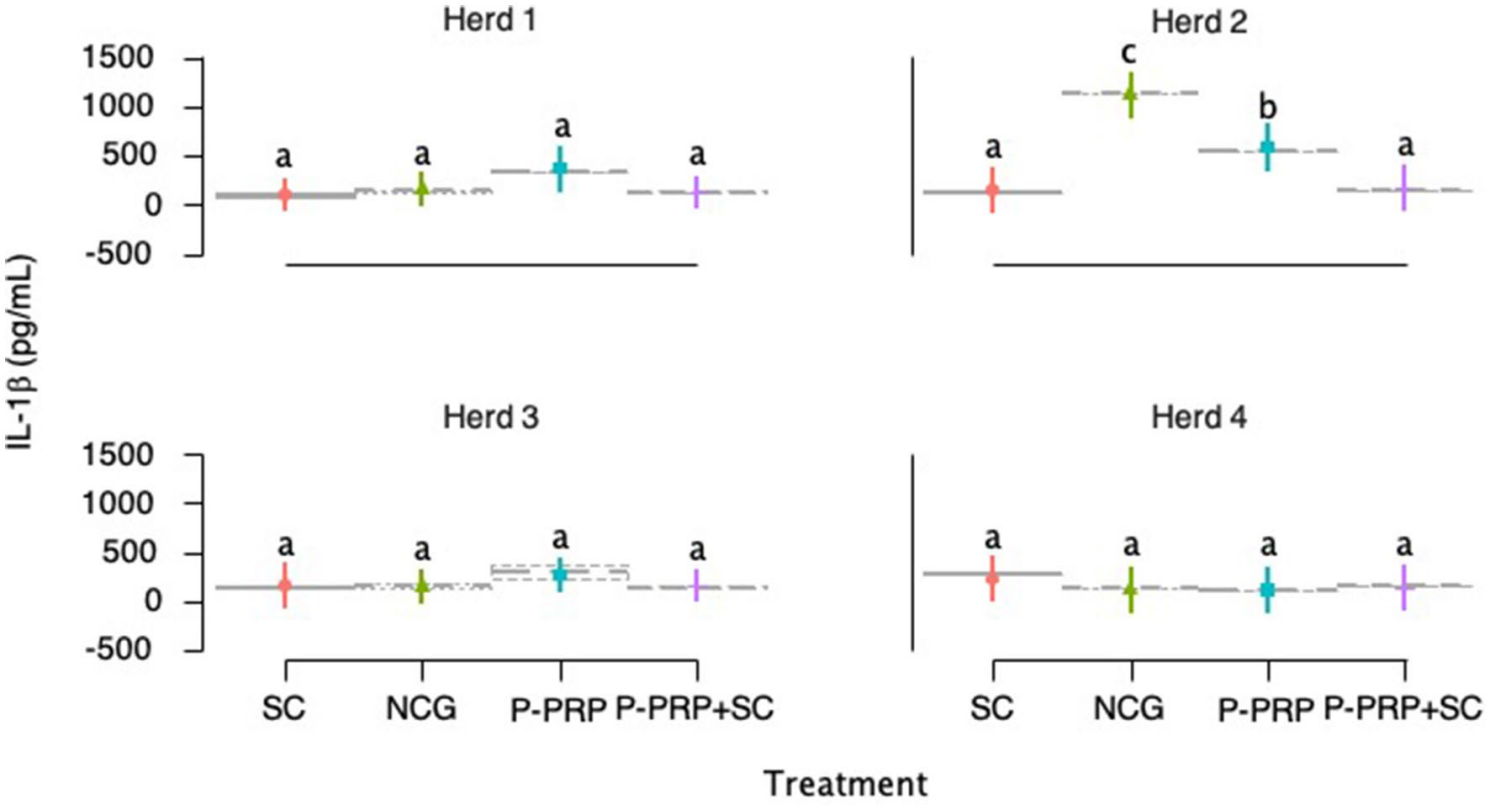
Box plots showing the confidence intervals (95%) for the mean concentrations (pg/mL) of interleukin 1 beta (IL-1β) in the milk of the study cows according to the interaction between treatment x farm factors. a-c= different lower-case letters represent statistically significant differences (<0.001) between the groups for the Scheffé test. Acronyms as in Tables 2 and 4.

The analysis of the interaction between treatment and farm factors showed that the cows treated with P-PRP and the animals from NCG of herd 2 had significantly (<0.001) higher concentrations of IL-1β than the cows of the other treatment groups of herds 1, 3, and 4. On the other hand, the milk concentrations of this inflammatory mediator were significantly higher in the NCG cows of herd 2 than in the P-PRP-treated animals of the same herd (Figure 7).

**Figure 8:**
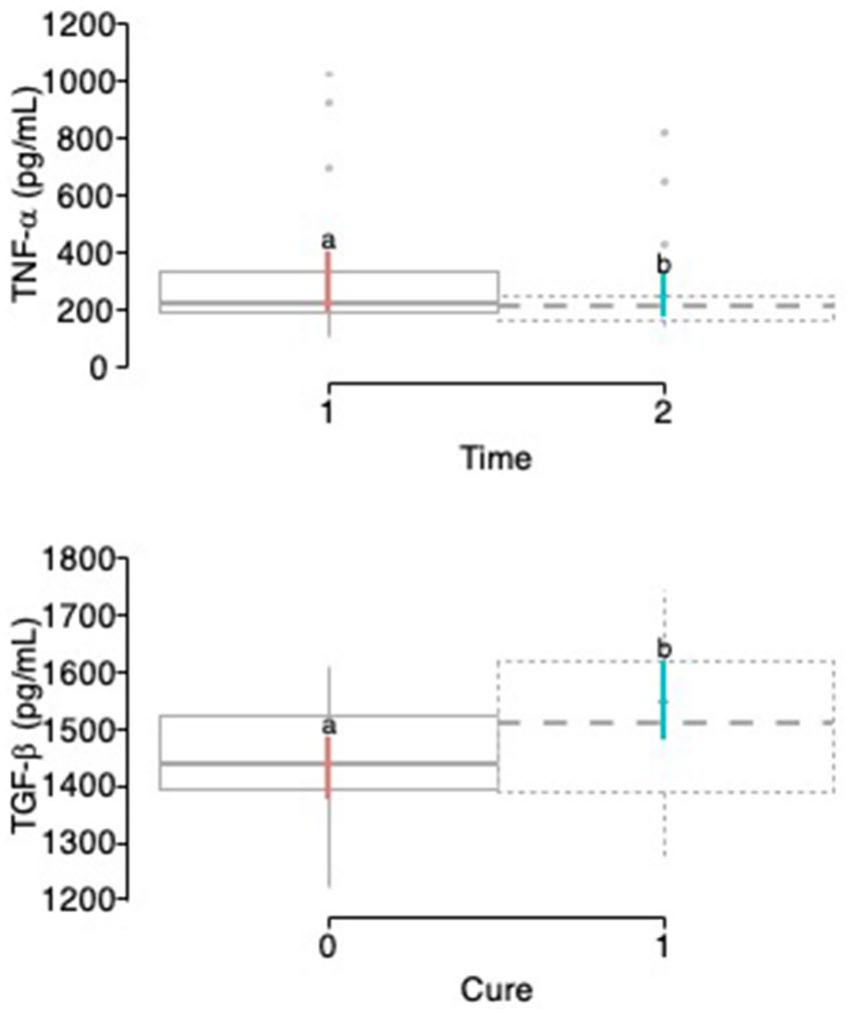
Box plots showing the confidence intervals (95%) for the mean concentrations (pg/mL) of tumor necrosis factor-alpha (TNF-α) **(A)** in the milk of the cows according to the time factor (1 = day 0; 2 = day 21), and transforming growth factor-beta 1 (TGF-β_1_) **(B)** in the milk of the cows according to the cure factor (0 = infected; 1 = cured). ^a,b^Different lower-case letters represent statistically significant differences (*p* < 0.05) between the groups for the Scheffé test. Acronyms as in Tables 2, 4.

Regarding the TNF-α milk concentrations, it was observed that in the exploratory model, the fixed factor time significantly influenced the concentration of this mediator, while other fixer factors evaluated did not influence the model (Table 11). Regarding the time factor, it was observed that TNF-α milk concentrations were significantly lower at 22 days than at baseline (Figure 8A).

**Table 11:** GLMMs evaluating the effect of fixed factors and their interaction on the necrosis tumor factor-alpha (TNF-α pg./mL) concentrations in the milk of the cows of the study.

GLMM type	Fixed factor	df	ChiSq	*p*
Exploratory	Intercept	1	22.742	< 0.001
	Treatment	3	4.734	0.192
	Time	1	19.304	< 0.001
	Herd	3	4.562	0.207
	Parity (n)	4	3.310	0.507
	LSSCC/mL	1	1.062	0.303
	Cure	1	0.001	1.000
	Bacteria group (BG)	1	0.005	0.941

Acronyms as in Tables 2–4.

In the exploratory model, TGF-β_1_ milk concentrations were significantly affected by the time and cure factors, while the other factors did not affect the model (Table 12). In the interaction model, it could be seen that the cure factor was the only fixed factor that significantly (*p* < 0.001) influenced the model (Table 12; Figure 8B).

**Table 12:** GLMMs evaluating the effect of fixed factors and their interaction on the transforming growth factor-beta 1 (TGF-b_1_pg/mL) concentrations in the milk of the cows of the study.

GLMM type	Fixed factor	df	ChiSq	*p*
Exploratory	Intercept	1	27.283	< 0.0001
	Treatment	3	0.000	1.000
	Time	1	81,359	< 0.001
	Herd	3	3,622	0.305
	Parity (*n*)	4	0.000	1.000
	LSSCC/mL	1	0.000	1.000
	Cure	1	14.146	< 0.001
	Bacteria group (BG)	1	0.000	1.000
Interaction	Intercept	1	172.443	< 0.001
	Time	1	0.000	1.000
	Cure	1	29.450	< 0.001
	Time × cure	3	0.000	1.000

Acronyms as in Tables 2–4.

## Discussion

The results of the present study provide novel information on the therapeutic potential of P-PRP and its combination with antibiotics, such as cloxacillin sodium, for the treatment of bovine SCM caused by major Gram-positive pathogens, such as *S. aureus*, *S. uberis*, and *S. dysgalactiae*. To the best of the author’s knowledge, the present research is probably the most comprehensive investigation to date that has examined the therapeutic role of an allogeneic hemocomponent technically categorized as a type of P-PRP (14).

The most important features of the present study are its methodological design, which included an adequate number of cows for each therapeutic group evaluated, as well as the inclusion of an NCG. These features allowed the development of an investigation with high statistical power and a low risk of bias. On the other hand, this study provides important information on the biological response of the mammary gland to the pathogens causing SCM and how it responds to the different treatments evaluated.

In line with the above, and although from the biological point of view, it is not possible to study a complete arrangement of cellular events and the production of soluble mediators in a dynamic event of bovine SCM, this research sought to know, in part, to know the behavior of some fundamental parts in this pathological process and against the therapeutic response generated in it by the effect of the treatments evaluated in the study. In this sense, it was decided to measure SCC in milk, as well as the concentration of IL-1β, TNF-α, and TGF-β_1_.

It is important to note that SSC is perhaps the best biomarker related to mammary gland health and one of the indicators of the immune response to mammary gland infection, which is why its constant monitoring is essential within the mammary health programs of a dairy herd (19). In general, elevated SCC above the established cutoff points for primiparous (<100 × 10^3^ SC/mL of milk) or multiparous (<200 × 10^3^ SC/mL of milk) cows are associated with subclinical infection of the mammary gland of cows, and their reduction over time is almost always associated with healing or resolution of such pathology (20, 21). On the other hand, the cytokines IL-1β and TNF-α are potent proinflammatory mediators that could reflect the degree of inflammation of the mammary tissue before and after the effect of the different treatments evaluated, while TGF-β_1_ is a pleiotropic growth factor with a potent anti-inflammatory and anabolic effect on cells and tissues (15, 22, 23).

To the best of the authors’ knowledge, this is the first clinical study to be conducted in crossbred dairy cows with SCM under tropical environmental conditions. As mentioned in the results section of this report, 30 cows belonging to five farms were excluded for various reasons that did not allow them to complete the study. This type of situation is one of the most common problems encountered by clinical researchers, and one of the ways to prevent it is to increase the number of animals treated per group to avoid the loss of statistical power of the study (24).

The concentration of platelets and leukocytes, as well as the concentration of TGF-β_1_, PDGF-BB, and PF-4 per mL of this hemocomponent, were within the concentrations previously described for this hemocomponent (14), which was previously evaluated in a clinical study of the same type (15). Typically, the hemocomponent evaluated in the present study corresponds to a P-PRP that has a similar or slightly increased concentration of platelets compared to the counts of these cytoplasmic fragments in whole blood from donor animals, as well as an extremely low or negligible concentration of leukocytes (25).

In the present study, 25% of the cows treated with P-PRP and 32.14% of the cows treated with SC showed a complete bacteriological cure. Comparing these results with our previous clinical study (15), a slight decrease in the efficacy of PRP of 5% and a drastic decrease in the therapeutic effect of the antibiotic of 38.36% can be seen. Regarding this last aspect, it should be clarified that in our first clinical study, we used a fourth-generation cephalosporin (cefquinome sulfate) (15), which is pharmacologically more potent against bacteria resistant to β-lactam antibiotics than SC (4, 26). However, despite the high therapeutic efficacy of cefquinome sulfate, drug regulatory agencies in some countries, and even the World Health Organization, have recommended that its use in farm animals be banned, arguing that such antibiotics should be kept in therapeutic reserve for use in humans to reduce potential microbial cross-resistance to antibiotics (27).

Consistent with the previous results, a contradictory fact was observed regarding the NCG, as 38.46% of the cows in this group showed spontaneous healing without the need to receive any treatment. The cows treated with P-PRP had a significantly lower probability of healing than the cows in the NCG. On this interesting aspect, it is possible to think that the use of this hemocomponent may cause a decrease in the immunological mechanisms of the udder and therefore decrease the probability of cure against an infection caused by *S. aureus* of this organ. However, this fact should be reserved until controlled experimental studies can be carried out in which mammary infections can be performed in homogeneous groups of cows with specific pathogens and of the same genetic pattern.

On the other hand, the most important result of our study was that the combination of P-PRP with SC produced a significant increase in the overall bacteriological cure (72.22%) in the group of cows with SCM that were treated with this therapeutic mixture. This result was significantly superior to the other groups evaluated in which a percentage difference of 47.22% was obtained vs. P-PRP, 40.08% vs. antibiotic, and 33.76% vs. NCG was obtained. It is worth mentioning that in a study where cows were treated with CM (acute and chronic), a similar synergistic therapeutic effect was observed (16).

It is possible that the P-PRP contained in this mixture enhanced the ability of the antibiotic to penetrate the bacterial cell walls or affected some metabolic or structural component of the bacteria to enable the microbicidal effect of the antibiotic (26, 28), even in bacterial strains resistant to it. However, this last aspect is a plausible hypothesis that should be addressed in future studies.

The results of the statistical models on the overall cure allowed us to demonstrate, in addition to the significant effect of treatment in the exploratory model, effects associated with the fixed-factors farm and LSSCC/mL at day 0 (29–31). The herd effect may influence the cure of these types of pathologies as the particular conditions of a herd may influence the results; the nutritional conditions of the cows, the sanitary management practices, and the environmental distribution of bacterial strains, among others, could be related to a particular herd (31).

In our case, we observed a lower cure response in cows from herd 1 than in animals from herds 2 and 3, while the overall bacteriological healing of herd 4 was similar to that of the other farms. At this point, it is necessary to carry out studies that will allow us to go deeper into the environmental conditions of these herds to improve the response to SCM treatments in their infected cows. On the other hand, it was noted that regardless of the treatment assigned, animals with LSSCC/mL at day 0 below 15.96 cells/mL were more likely to show bacteriological cure than cows with higher LSSCC/mL. This latter finding has been reported in studies in which *S. aureus* SCMs were produced (1, 20, 31).

Individual analysis of the cure of *S. aureus*-induced SCM in the groups of cows treated with P-PRP, SC, P-PRP + SC, and those that were not treated (NCG) was 25, 29.2, 44.4, and 50%, respectively. Unfortunately, these results indicate that no therapy was effective in managing this disease, and even untreated cows could clear the infection just as well without incurring treatment costs for these producer-reported infections (1, 20, 31). On the other hand, it was observed that cows with a day 0 LSSCC of less than 14.25 cells/mL were significantly more likely to be cured than cows with higher counts of these cells. It is important to clarify that one of the main causes of culling in dairy cows is clinical and subclinical *S. aureus* infections, which are often refractory to conventional antibiotic therapies (32, 33), as at the mammary level this type of bacteria can colonize the interior of mammary epithelial cells (34). Based on the present results, it is necessary to develop an additional clinical study to determine the degree of antibiotic resistance of the individualized strains of these bacteria for each cow to be treated, so that only antibiotics with an effective therapeutic probability are used and to determine their potential synergistic effect against mammary infections caused by these bacteria and how the PRP could improve the cellular penetration capacity of the antibiotic (34).

Regarding the SCM caused by the group of streptococci (*S. uberis* and *S. dysgalactiae*), the cure was observed in 33.3% of the cows treated with P-PRP, 50% of the animals treated with SC, 90% of the cows treated with the mixture of P-PRP + SC, while the cows of the NCG presented a spontaneous cure in 25% of the cases. It must be clarified that the treatment was the only fixed factor influencing the cows to be cured. The results of the present study, especially for the P-PRP, were lower (33%) than those obtained for the same hemocomponent in a previous study, where 50% of the cows infected with this group of pathogens showed a bacteriological cure (15). In addition, a proportion of cows treated with SC also had a lower cure rate (50%) than cows treated with cefquinome sulfate (90%) (15).

However, the combination of SC and P-PRP had a similar therapeutic effect (90%) as cefquinome sulfate. This last finding is encouraging because P-PRP may enhance the effect of b-lactam antibiotics on bacteria that appear to be resistant to them. It is important to note that PRP did not reduce the amount of SC needed to produce healing in cows treated with this combination. However, it seems that this hemocomponent acted synergistically, potentiating the bactericidal action of SC.

As mentioned above, the innate defense capacity of the mammary gland, particularly related to the leukocytes (somatic cells) excreted through the milk, represents one of the most important facilitating mechanisms for the resolution of clinical and subclinical mammary infections and, as previously observed, for the resolution of SCMs produced by *S. aureus*. In our study, cured cows were observed to have significantly lower LSSCC/mL than uncured cows, regardless of the treatment used. This finding is consistent with previous reports showing that animals with low SCC are more likely to be cured of episodes of SCM and CM than animals with high SCC (35, 36).

Both IL-1b and TNF-a could be considered as the most important pro-inflammatory mediators in various pathological processes, including inflammatory processes of infectious origin, such as SCM (34, 37, 38). These cytokines are activated through the nuclear factor kappa B pathway or activating protein 1 (AP-1) and, once released, can self-perpetuate inflammation by increasing the expression of the aforementioned pro-inflammatory pathways (34, 39–41).

In the present study, it was observed that the concentrations of IL-1β in the milk were significantly influenced by the treatment factor, and in particular, cows treated with SC or with the mixture of P-PRP + SC had the lowest concentrations of this mediator concerning the NCG. On the other hand, a significant effect of herd 2 was also observed in terms of increased milk concentrations of this cytokine compared to the other farms. On this point, it is difficult to establish the causes associated with this increase, but a possible explanation for it could be related to zootechnical or hygienic factors of the same, which could be different compared to the other dairy herds, or to the fact that most of the cows in the NCG came from this dairy farm.

Regarding TNF-a, it was observed that this cytokine was only affected by time, a fact that could indicate a low value for the diagnosis or prognosis of SCM in cows as milk concentrations of this cytokine were not associated with the healing of SCM or its response to treatment. The present results contradict some *in vitro* studies that have shown that this cytokine is key in inducing apoptosis of *S. aureus*-infected cells and thus promoting resolution of the infection (34, 42).

In the present study, we were able to observe that TGF-b_1_ milk concentrations were significantly affected by the cure factor. Cows that were cured of the infection, regardless of the treatment used, had significantly higher concentrations of this mediator than cows that were not cured. Our results are inconsistent with other studies that have failed to demonstrate a definitive role of this cytokine in the pathophysiological process of SCM (22) or that have observed that the increase in this cytokine could be associated with the exacerbation of *S. aureus* infection (43, 44). At this point, and considering the results of the present study, it is possible to think that TGF-b_1_ could act as an anti-inflammatory and anabolic cytokine that could be key to the resolution of natural subclinical infections of the mammary gland produced by *S. aureus* and streptococci (45).

The clinical trial had several limitations. One was the fact that the cows were only followed for 22 days. This situation limited the ability to know the exact recurrence rate of infection for each specific treatment. In addition, antibiograms were not performed to know how many of the cows treated with SC were susceptible to this antibiotic and whether the addition of P-PRP could increase bacterial susceptibility to SC in bacteria previously resistant to this antibiotic. Further studies are needed to ensure long-term follow-up of the cows and to document the antimicrobial susceptibility of the bacteria causing infection in the cows enrolled.

## Conclusion

The results of the present study provide new data on the effect of the combination of P-PRP and SC as a treatment for bovine SCM, especially that caused by non-agalactiae streptococci, where the mixture of both substances produced a cure in 90% of the treated cases. It is important to note that treatment was the only way to resolve this type of infection in the cows in the study.

On the other hand, *S. aureus*-induced SCM continues to be a major therapeutic challenge since in general none of the treatments evaluated showed an effective therapeutic response and only the specific immunological conditions of the cows, i.e., having low milk SCC counts at the beginning of the study, represent aspects of the innate immune system that facilitate the healing of SCM induced by this bacterium without the need for any treatment.

Further controlled studies, both clinical and experimental, are needed to improve P-PRP as a single treatment or as an antibiotic mixture for the treatment of SCMs produced by Gram-positive bacteria.

## Data Availability

The raw data supporting the conclusions of this article will be made available by the authors, without undue reservation.
